# Guardians and the Mental Deficiency Act

**Published:** 1914-02-21

**Authors:** 


### THE CENTRAL POOR-LAW CONFERENCE.

### Guardians and the Mental Deficiency Act.

The forty-second annual Central Poor-Law Conference of the Poor-Law Guardians of England and Wales was held at the Guildhall on Tuesday and Wednesday last week. Lord Methuen presided.

### The Care of Mental Defectives.

Mr. R. J. Curtis, Clerk of the Birmingham Union, read a paper on "The Poor Law and the Care and Training of Mentally Defective Persons." He said that such a question transcended all the interests of political party, creed and denomination, and reached to the very element of our common humanity. A new period had been ushered in by the passing of the Mental Deficiency Act, which set up a Board of Control as the central authority. That board had the difficult task of establishing a settled plan of action between the various authorities and agencies dealing with mental defects, so as to secure that such persons should not pass from one authority or institution to another, helped or detained a little at each, but permanently cared for by none. As the Act preserved the present powers and duties of existing authorities dealing with mentally defectives, it was difficult to see how overlapping and confusion could be avoided, but doubtless the Board of Control would do their utmost to evolve some sort of order. The ancient duty of Guardians to maintain the mental defectives chargeable was preserved, but it had to be regretfully acknowledged that despite their persistent efforts they had failed to secure from Parliament power (other than that contained in the Lunacy Act) to detain those classes of feeble-minded persons who were known to be a danger to themselves and the community. Strong efforts to that end were made during the passing of the Bill, but the Parliamentary Committee refused to grant the power, even in respect of premises approved by the Board of Control, a refusal due to the spirit of antipathy to Boards of Guardians which pervaded both sides of the House. The new Act, therefore, left them in this position, that there were a very large number of mentally defective cases which the County Councils could deal with, either by providing institutions or by entering into an arrangement with Boards of Guardians; that in respect of the cases dealt with by Guardians no grant would be received, but that the County Councils would receive grants in respect of cases they dealt with, while the County Councils would have powers of detention which the Guardians would not. It seemed to him that the Guardians had not been well treated by the Government in this matter.

### The Need of Co-operation.

Mr. Curtis strongly urged the desirability of full co-operation between Boards of Guardians and the new local authority, and the Chairman of the Board of Control (Sir William Byrne) had authorised him to say that they would welcome such active co-operation. There were several ways in which that could be done. The local authority should make full use of its power to appoint Poor-Law Guardians upon the new committee. A workhouse could be used as a place of safety for defectives, and Guardians could assist by not placing any obstacle in the way of this being done. The Board of Control might approve of buildings or premises provided by a Board of Guardians (or a Joint Committee) for the reception of defectives, and so long as the premises continued to be approved the Guardians and any local authority, not necessarily for the same area, might enter into agreements as to the reception and maintenance of defectives. It was to be hoped that those Guardians who possessed premises which could with reason be said to be specially fit for both the care and training of defectives would not hesitate to offer the same for approval. It had already been officially announced that it would be useless to seek the authority of the board for the use of ordinary workhouse accommodation for the detention of defectives, and applications would be favourably entertained only when the premises in question had been specially provided. Possibly some Boards could assist the ratepayers by effecting a contract under Section 26 of the Lunacy Act and housing harmless certified patients, thus releasing the ever-increasing pressure upon county mental hospitals.

### The Colony and Combination.

Very few Unions had a sufficient number of cases to enable them acting alone to provide a colony or an institution in which to classify satisfactorily the varying grades of cases that admittedly should be provided for in institutions specially adapted for their care and training. There was no sound reason why Guardians should hesitate to combine; on the contrary, there was every reason why they should do so and without delay. Up to the present forty-four Unions had combined: in seventy-nine cases orders were expected, and in twenty negotiations were practically completed, and various Unions in at least fifteen other counties were giving attention to the subject.

The lack of full care and of training of those suitable was costly in Poor-Law relief, in voluntary charity, in asylum maintenance, and in expenditure on police and prisons, for there was clear evidence that the criminal classes were chiefly recruited from mentally defectives. The new Act would tend to bring against the evil all the forces of several administrative agencies, and as one of these Guardians should adopt a progressive policy and play an indispensable part. He illustrated his remarks by a description of the Monyhull Colony for Epileptics at Birmingham, the work of which is already known to our readers.

### The Discussion.

Sir H. J. Manton, Birmingham, said that Guardians had largely themselves to blame for the position they found themselves in. The Conference had for years past urged them to bury their mutual jealousies and enter into combinations. He did not like to see the County Councils doing this work, because the atmosphere of an asylums committee was not the one they wanted for the mentally deficient. It was the weakness of the Guardians' voice that had prevented Parliament acceding to their demands.

Mr. W. Murray, Tynemouth, urged that it would be much better if they could eliminate the words "Poor Law" and "Guardian."

Mr. Galleon, Cannock, declared that no work of the County Councils was more criticised than their extravagant dealing with mental cases. The Guardians were far better able to deal with imbeciles than the County Councils. Every Union in Staffordshire had joined, or was attempting to join, a combination.

A summarised report of the second day's proceedings will appear in The Hospital next week.

